# Clinicopathological features and prognosis of patients with gastric neuroendocrine tumors: A population‐based study

**DOI:** 10.1002/cam4.1683

**Published:** 2018-10-11

**Authors:** Fang‐Xi Tian, Yu‐Qing Cai, Lv‐Ping Zhuang, Ming‐Fang Chen, Zhong‐Biao Xiu, Yi Zhang, Hong Liu, Zhi‐Hong Liu, Guo‐Ping Liu, Chen Zeng, Fei‐Ling Lin, Jing Liu, Si‐Ting Huang, Liang‐Zhi Zhang, Hua‐Yang Lin

**Affiliations:** ^1^ Department of Gastric Surgery Fujian Medical University Union Hospital Fuzhou China; ^2^ Fujian Medical University Union Hospital Fuzhou China; ^3^ Zhejiang University School of Medicine Hangzhou China; ^4^ Fujian University of Traditional Chinese Medicine Fuzhou China; ^5^ The Studio of Acupotomology The Affiliated People's Hospital of Fujian University of Traditional Chinese Medicine Fuzhou China; ^6^ Department of Anesthesiology The Affiliated People's Hospital of Fujian University of Traditional Chinese Medicine Fuzhou China

**Keywords:** conditional survival, dynamics, gastric neuroendocrine tumor, prognosis

## Abstract

**Background:**

Despite its rarity, studies have shown the incidence of gastric neuroendocrine tumors (G‐NETs) is increasing. This study investigated the risk factors affecting the survival of G‐NETs patients and their prognosis over time.

**Method:**

A retrospective analysis of 506 G‐NETs patients who underwent surgery for nonmetastatic disease from the Surveillance, Epidemiology and End Result database from 1988 to 2011 was conducted. Multivariate Cox regression analyses identified the prognostic factors affecting overall survival (OS) and disease‐specific survival (DSS). Three‐year conditional survival (COS3 and CDS3) estimates at “*x*” year after treatment were calculated as follows: COS3 = OS(*x* + 3)/OS(*x*) and CDS3 = DSS(*x* + 3)/DSS(*x*).

**Results:**

The 1‐, 3‐, and 5‐year OS rates of all patients after surgery were 90.2%, 77.3%, and 68.8%, respectively. The 1‐, 3‐, and 5‐year DSS rates after surgery were 93.9%, 84.5%, and 80.9%, respectively. In the multivariate analysis, age, tumor grade, and T stage were independent prognostic factors of OS and DSS (all *P* < 0.05). With 1‐, 3‐, and 5‐year survivorship, the COS3 improved by +5.2 (82.2%), +7.2 (84.4%), and +8.5 (85.5%), respectively, and the CDS3 improved by +4.4 (89.4%), +9.1 (94.1%), and +12.5 (97.5%), respectively. Notably, the CDS3 improved dramatically among patients with advanced stage disease (eg, N0 stage: 93.0%‐98.9%, Δ5.9% vs N1 stage: 52.0%‐95.7%, Δ43.7%).

**Conclusion:**

For G‐NETs patients, age, tumor grade, T stage, and N stage were the clinicopathological factors significantly associated with prognosis. There were excellent outcomes for most G‐NETs patients, with a CDS3 of greater than 90% across all independent prognostic factors after 5 years of survival.

## 1 INTRODUCTION

Neuroendocrine tumors (NETs) are a type of tumor derived from the diffuse neuroendocrine system, and they account for 1%‐2% of malignant tumors.^1^ Gastric neuroendocrine tumors (G‐NETs) are rare, with prevalences of 3.2 and 1.7 per 100 000 people in European countries and the United States, respectively.^2^

Based on the current epidemiological data, the worldwide incidence of NETs has seemed to increase.^2^, ^3^, ^4^, ^5^ The greatest increase in incidence occurred for gastric and rectal NETs.^6^, ^7^ The rising incidence of G‐NETs over time may be attributed to factors such as increased clinical and pathological experience in diagnosing this disease, heightened physician awareness, and increased endoscopic surveillance.^8^, ^9^

Given their rarity and increasing prevalence, understanding the natural history and long‐term outcomes of G‐NETs is essential for clinicians so that they may best supervise their patients. However, due to significant differences in the biological characteristics, our knowledge of G‐NETs is still very limited. In addition to an early diagnosis, an important and effective component of proper management is the identification of prognostic factors in patients with G‐NETs. The European Neuroendocrine Tumor Society (ENETS) TNM staging system,^10^ which accounts for invasion depth, lymph node status, and metastases, is one of the most important prognostic factors in patients with G‐NETs. However, owing to its low incidence, research on the clinicopathological features and prognosis of patients with G‐NETs is lacking.

Conditional survival (CS) estimates may be a more useful way to predict long‐term prognosis than conventional survival estimates, given that survival probabilities can change significantly when accounting for time elapsed after treatments.^11^, ^12^, ^13^ This concept has been confirmed in various cancers, including lung cancer, thyroid cancer, gastric cancer, colorectal cancer, and pancreatic carcinoma.^12^, ^13^, ^14^, ^15^, ^16^ To our knowledge, there has been no previous study assessing CS among patients with G‐NETs.

Therefore, we aimed to identify the prognostic factors of overall survival (OS) and disease‐specific survival (DSS) among patients with G‐NETs in a large population‐based database. Moreover, we also assessed the prognosis of surgically resected G‐NETs patients with prolonged survival times.

## 2 METHODS

### 2.1 Patient population and data collection

As a population‐based cancer registry that collects cancer incidence and survival data from 18 regional population‐based registries, the Surveillance, Epidemiology and End Result (SEER) database covers approximately 27.8% of the US population (based on the 2010 census).^17^ The Site and Morphology of Collaborative Stage Data Collection System (CS Schema v0204+) was used to identify GEP‐NET cases.^18^ The code of NETStomach was used for the identification of the histological type and tumor location of G‐NETs. We included patients who underwent surgical resection from the SEER database from 1988 to 2011 based on the following characteristics: microscopic confirmation of the tumor, the presence of single primary tumor, the availability of complete staging information, and survival for more than 1 month. The selection scheme using the SEER database is shown in Figure S1.

Sociodemographic and clinicopathologic data were routinely obtained. The tumor sites were grouped into four subsites as follows: proximal third (C16.0 and C16.1); middle and distal third (C16.2, C16.3, and C16.4); stomach, not otherwise specified (NOS) (C16.5, C16.6, and C16.9); and overlapping (C16.8). We set the size (the longest diameter) of 20 mm as a segmentation point based on the NCCN Clinical Practice Guidelines.^19^ Notably, the SEER grading system classifying tumors into well differentiated (SEER grade 1), moderately differentiated (SEER grade 2), poorly differentiated (SEER grade 3), and undifferentiated/anaplastic (SEER grade 4) relies on histologic differentiation, which is different from the 2010 WHO grading nomenclature. Therefore, we combined SEER grade 3 and 4 data into “grade 3 neuroendocrine carcinomas,” as previously reported.^1^, ^20^, ^21^ T stage and N stage were classified according to the criteria described in the ENETS consensus.^10^ Socioeconomic status data (based on the 2010 census) were collected, including the percentage of people with less than high school education, percentage of families in poverty, percentage of unemployed people, percentage of foreign‐born people, percentage of families in language isolation, and median household income. Patients in the study cohort were divided into two categories separated by the median value for each of these attributes (Table S1).

### 2.2 Statistical methods

The cause of death for SEER cohorts was defined using the SEER cause of death codes.^17^, ^22^ Deaths from G‐NETs were coded as disease‐specific mortality.

The association of relevant clinicopathologic variables with OS and DSS was assessed using a Cox proportional hazards model. Variables that were statistically significant in univariable analysis (*P* < 0.05) were retained in the multivariable model.

The CS is originally derived from conditional probability in biostatistics, and it can be calculated using the life‐table method.^15^ The CS3 at *x* years means the probability of an additional 3 years of survivorship in a patient who has already survived for *x* years after the initial treatments, and it is calculated as follows: CS3 = *S*(*x* + 3)/*S*(*x*).^11^

In the current study, the data of OS and disease‐specific survival were used to calculate 3‐year conditional overall survival (COS3) and 3‐year conditional disease‐specific survival (CDS3), respectively. Moreover, significant variables correlated with survival time in the Cox proportional hazards model were used for the COS3 and CDS3 calculations. The difference in CS among groups was compared via the standardized differences (*d*) method, which was first described by Cucchetti et al^23^ and has been subsequently employed by several groups.^24^, ^25^ The *d* value was calculated as follows: *d* = (*P*2 − *P*1)/√[*P*(1 − *P*)]. |*d*| < 0.1 indicates very small differences among groups, 0.1 ≤ |*d*|<0.3 indicates small differences, 0.3 ≤ |*d*|<0.5 indicates moderate differences, and |*d*|≥0.5 indicates clear differences.

Categorical data were summarized with frequencies and percentages. All the data were processed using SPSS 19.0 (SPSS Inc. Chicago, IL, USA) and R software (version 3.4.3) (https://cran.r-project.org/). All the tests were two‐sided with the significance level set to *P* < 0.05.

## 3 RESULTS

### 3.1 Demographic and clinicopathologic characteristics

This retrospective study included 506 patients with G‐NETs who had detailed clinicopathological data. The mean age of the study population was 60 ± 13.8 years, and 54.9% of the patients were female. Most of patients were non‐Spanish‐Hispanic‐Latino (81.0%). According to the pathological findings, the number of patients with high‐grade tumors was 4 times as large as the number of those with low‐grade tumors (G1‐G2: n = 399, 78.9% vs G3: n = 107, 21.1%). Based on the ENETS staging system, 24.3% of patients had advanced T stage disease (T3‐T4: n = 123), and 20.6% of patients were found to have lymph node metastasis (N1: n = 104). Of the 506 patients, 12.3% received chemotherapy, and only 6.3% of patients received radiation.

### 3.2 Actual OS and DSS

**Figure 1:**
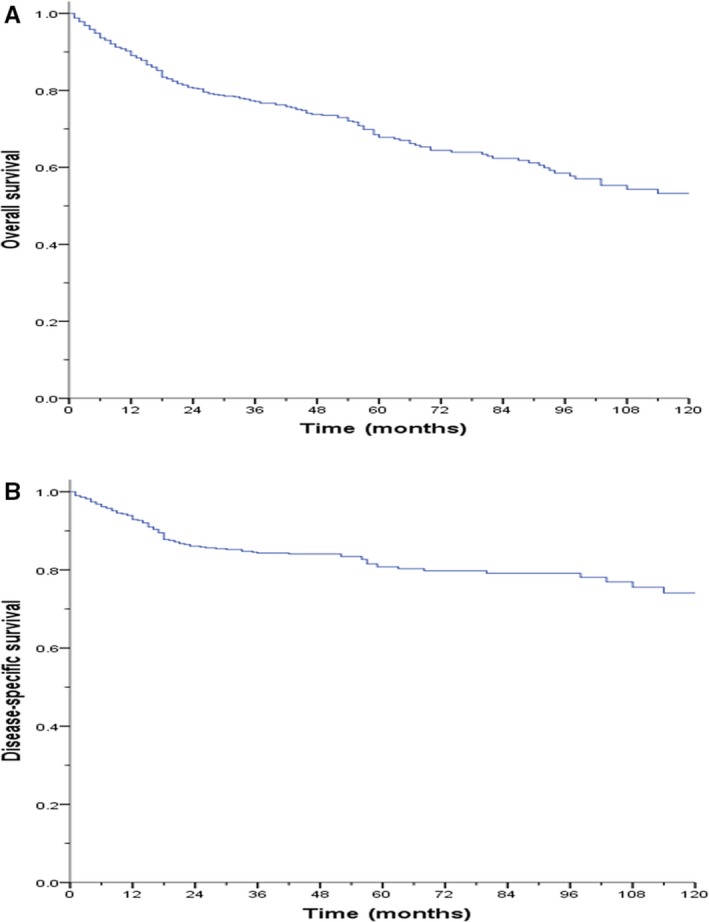
Kaplan‐Meier survival curve of overall survival (A) and disease‐specific survival (B) for the entire cohort

After a median follow‐up of 64.0 months (1‐235 months), 177 (35.0%) patients died, including 52% (n = 92) who died of disease‐specific causes. The 1‐, 3‐, and 5‐ year OS rates of all patients after surgery were 90.2%, 77.3%, and 68.8%, respectively (Table 1). The 1‐, 3‐, and 5‐ year DSS rates after surgery were 93.9%, 84.5%, and 80.9%, respectively (Table S3). Univariate analysis showed that several factors were related to OS (Table S2), including age, sex, ethnicity, tumor size, tumor site, tumor grade, T stage, N stage, and use of chemotherapy and radiation (all *P* < 0.05). Meanwhile, all the above factors were also related to DSS in univariate analysis (all *P* < 0.05). After adjusting for confounding factors (Table 2), older age (age ≥ 65 vs < 65, HR = 3.41, 95% CI: 2.48‐4.70; *P* < 0.001), high‐grade (G3 vs G1‐G2, HR = 2.62, 95% CI: 1.75‐3.93; *P* < 0.001), advanced T stage (T3‐T4 vs Tis‐T2, HR = 1.52, 95% CI: 1.02‐2.24; *P* = 0.038), and lymph node metastasis (N1 vs N0, HR = 1.44, 95% CI: 1.01‐2.07; *P* = 0.011) were independent risk factors for OS in multivariate analysis. Furthermore, the above factors were also independently associated with DSS (Figure 1). The changes in the actual OS and DSS of each independent prognostic factor within 8 years after surgery are shown in Tables S3 and S4.

**Table 1:** Sociodemographic and clinicopathologic variables of gastric neuroendocrine tumors patients (n = 506)

Variable	No. of patients	%	Variable	No. of patients	%
Demographic			Tumor presentation		
Age y			Primary site		
Mean ± SD	60.0 ± 13.8		Proximal	101	20.0
<65	309	61.1	Middle and distal	194	38.3
≥65	197	38.9	Overlapping lesions	20	4.0
Sex			Stomach, NOS	191	37.7
Female	278	54.9	Size, mm		
Male	228	45.1	≤20	288	56.9
Race			>20	139	27.5
White	399	78.9	Unknown	79	15.6
Black	66	13.0	Grade		
Others	34	6.7	G1‐G2	399	78.9
Unknown	7	1.4	G3	107	21.1
Ethnicity			ENETS T stage		
Spanish‐Hispanic‐Latino	96	19.0	Tis‐T2	383	75.7
Non‐Spanish‐Hispanic‐Latino	410	81.0	T3‐T4	123	24.3
Socioeconomic			ENETS N stage		
Marital status			N0	402	79.4
Unmarried	183	36.2	N1	104	20.6
Married	294	58.1	ENETS staging		
Unknown	29	5.7	0	68	13.4
Education			I	162	32.0
Advantaged	247	48.8	Treatment		
Disadvantaged	259	51.2	Chemotherapy		
Poverty			No	444	87.7
Advantaged	253	50.0	Yes	62	12.3
Disadvantaged	253	50.0	Radiation		
Unemployment			No	474	93.7
Advantaged	259	51.2	Yes	32	6.3
Disadvantaged	247	48.8	
Family income		
Advantaged	252	49.8
Disadvantaged	254	50.2
Foreign‐born		
Advantaged	251	49.6
Disadvantaged	255	50.4
Language isolation		
Advantaged	251	49.6
Disadvantaged	255	50.4

ENETS, The European Neuroendocrine Tumor Society; NOS, not otherwise specified; SD, standard deviation; Unmarried, single (never married), separated divorced, widowed, unmarried or domestic partner (same sex or opposite sex or unregistered); Y, year.

**Table 2:** Multivariate analysis of prognostic factors of overall survival and disease‐specific survival for gastric neuroendocrine tumor patients

Variable	Overall survival			Disease‐specific survival		
	Multivariate model			Multivariate model		
	HR	95% CI	*P*	HR	95% CI	*P*
Age, y			<0.001			<0.001
<65	Ref.			Ref.		
≥65	3.41	2.48‐4.70	<0.001	2.24	1.45‐3.48	<0.001
Sex			0.114			0.760
Female	Ref.			Ref.		
Male	1.29	0.94‐1.76	0.114	1.07	0.69‐1.67	0.760
Primary site			0.525			0.272
Proximal	Ref.			Ref.		
Middle and Distal	0.76	0.51‐1.14	0.183	0.73	0.43‐1.30	0.256
Overlapping lesions	0.79	0.37‐1.69	0.549	0.97	0.40‐2.37	0.943
Stomach, NOS	0.76	0.51‐1.14	0.186	0.59	0.35‐1.02	0.059
Size, mm			0.284			0.646
≤20	Ref.			Ref.		
>20	1.27	0.84‐1.92	0.257	1.32	0.67‐2.57	0.421
Unknown	1.41	0.91‐2.20	0.124	1.40	0.67‐2.92	0.369
Grade			<0.001			<0.001
G1‐G2	Ref.			Ref.		
G3	2.62	1.75‐3.93	<0.001	5.91	3.14‐11.13	<0.001
ENETS T stage			0.038			0.002
Tis‐T2	Ref.			Ref.		
T3‐T4	1.52	1.02‐2.24	0.038	2.56	1.41‐4.65	0.002
ENETS N stage			0.450			0.006
N0	Ref.			Ref.		
N1	1.44	1.01‐2.07	0.450	1.98	1.22‐3.22	0.006
Chemotherapy			0.635			0.473
No	Ref.			Ref.		
Yes	1.13	0.68‐1.87	0.635	1.22	0.71‐2.13	0.473
Radiation			0.484			0.427
No	Ref.			Ref.		
Yes	1.22	0.70‐2.11	0.484	1.26	0.71‐2.24	0.427

ENETS, The European Neuroendocrine Tumor Society; NOS, not otherwise specified; y, year.

### 3.3 COS and comparison with actual OS

**Figure 2:**
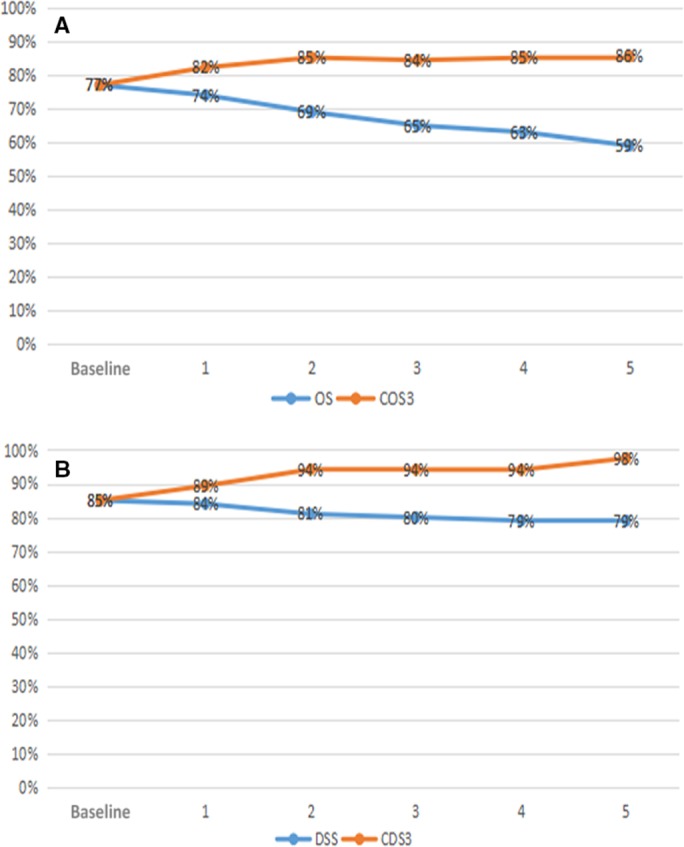
A, Conditional overall survival relative to actual overall survival; B, Conditional disease‐free survival relative to actual disease‐free survival

**Figure 3:**
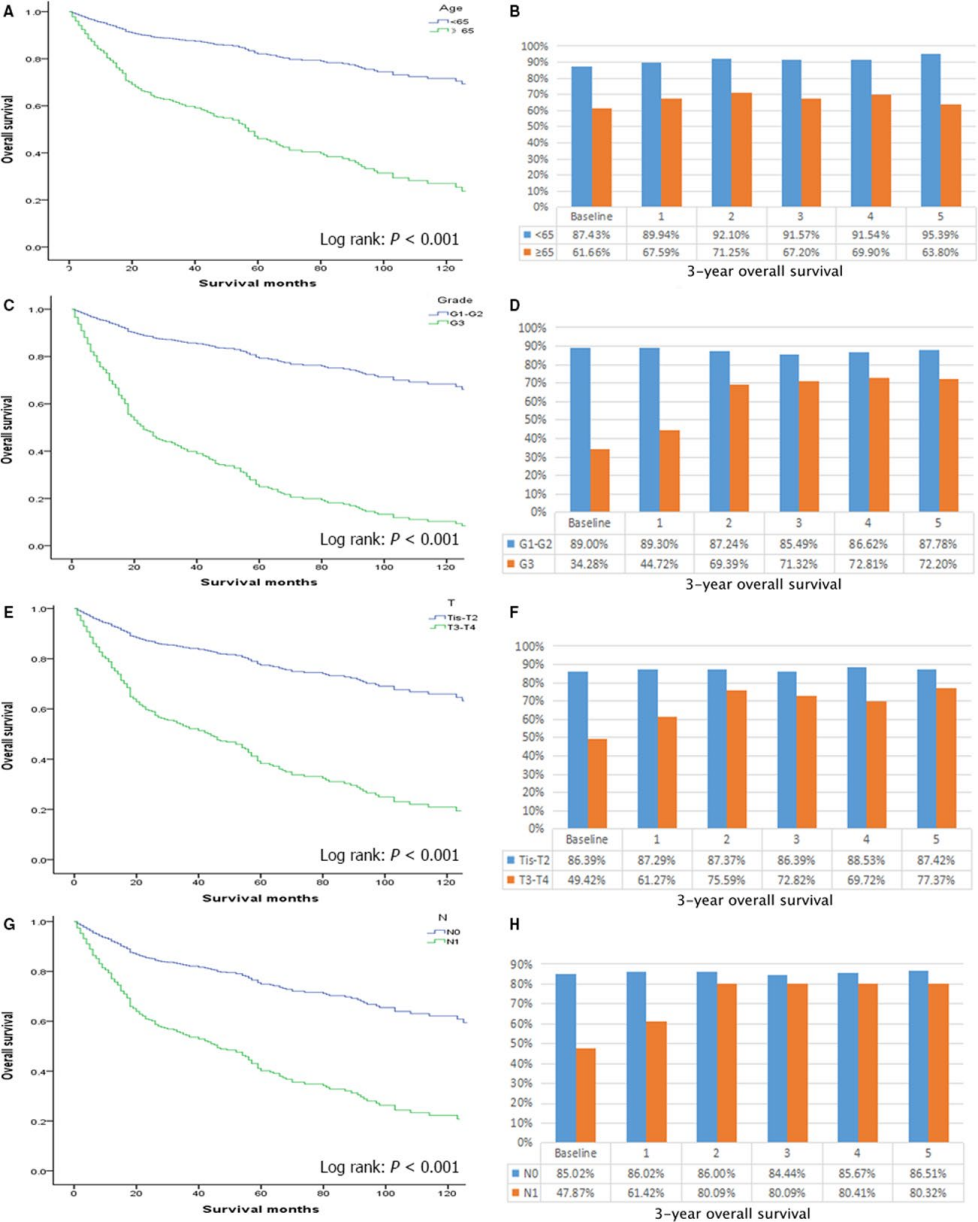
Actual overall survival stratified by: (A) age, (C) tumor grade, (E) ENETs T stage, and (G) ENETs N stage vs conditional overall survival relative to actual survival stratified by: (B) age, (D) tumor grade, (F) ENETs T stage, and (H) ENETs N stage

The Figure 2A shows the change in annual COS3 and actual OS within 5 years for all patients after surgery. The actual 3‐year OS rates were equal to the COS3 at the baseline. The COS3 improved with increasing survival time after surgery, while the actual OS had a downward trend. Given 1‐, 3‐, and 5‐year survivorship, the COS3 improved by +5.2 (82.2%), +7.2 (84.4%), and +8.5 (85.5%), respectively. The effects of the prognostic factors on the actual and conditional OS were assessed by the subgroup analysis of the patients. (Figure 3). According to the stratified analysis of each subgroup, the gap between the actual OS and COS3 became increasingly significant for patients with relatively poor initial prognoses. For example, the 8‐year OS among patients with low‐grade (G3) tumors was only 19.6%, and the COS3 after 5 years of survival reached 94.6% (Δ52.6%). However, the 8‐year actual OS for patients with high‐grade (G1‐G2) tumors was 70.5%, and the COS3 at 5 years was 87.8% (Δ17.3%). We also found that except for age, the differences in COS3 between the strata of each subgroup decreased over time (Table 3). For example, the differences in COS3 between T3‐4 stage and Tis‐T2 stage decreased from clear differences (|*d*| at the baseline = 0.88) at the baseline to moderate differences (|*d*| at 3 years = 0.36) at 3 years and small differences (|*d*| at 5 years = 0.28) at 5 years after surgery.

**Table 3:** Three‐year conditional survival rates of patients with gastric neuroendocrine tumors in relation to prognostic factors

Characteristic	COS3						CDS3					
	Years since diagnosis						Years since diagnosis					
	Baseline	1	2	3	4	5	Baseline	1	2	3	4	5
Overall	77.0%	82.2%	85.2%	84.4%	85.1%	85.5%	85.0%	89.4%	94.2%	94.1%	94.0%	97.5%
Age, y												
<65	87.4%	89.9%	92.1%	91.6%	91.5%	95.4%	90.0%	92.8%	97.8%	96.7%	95.6%	96.6%
≥65	61.7%	67.6%	71.2%	67.2%	69.9%	63.8%	75.0%	83.1%	85.9%	89.3%	90.5%	100.0%
d(<65 vs ≥65)	0.62	0.57	0.57	0.64	0.58	0.84	0.41	0.31	0.47	0.30	0.20	‐0.24
Grade												
G1‐G2	89.0%	89.3%	87.2%	85.5%	86.6%	87.8%	97.0%	98.0%	96.9%	96.9%	95.9%	97.9%
G3	34.3%	44.7%	69.4%	71.3%	72.8%	72.2%	38.0%	49.3%	73.8%	78.9%	83.3%	96.8%
d(G1‐G2 vs G3)	1.31	1.11	0.48	0.37	0.37	0.43	1.63	1.48	0.85	0.71	0.50	0.07
ENETS T stage												
Tis‐T2	86.4%	87.3%	87.4%	86.4%	88.5%	87.4%	95.0%	96.0%	96.9%	97.9%	97.9%	100.0%
T3‐T4	49.4%	61.3%	75.6%	72.8%	69.7%	77.4%	52.0%	64.6%	80.4%	82.7%	80.4%	91.1%
d(Tis‐T2 vs T3‐T4)	0.88	0.66	0.33	0.36	0.51	0.28	1.19	0.98	0.64	0.65	0.72	0.61
ENETS N stage												
N0	85.0%	86.0%	86.0%	84.4%	85.7%	86.5%	93.0%	94.9%	95.7%	95.7%	95.7%	98.9%
N1	47.9%	61.4%	80.1%	80.1%	80.4%	80.3%	52.0%	66.2%	85.5%	86.5%	88.2%	95.7%
d(N0 vs N1)	0.89	0.63	0.16	0.12	0.15	0.17	1.14	0.92	0.42	0.38	0.32	0.24

CDS3, 3‐year conditional disease‐specific survival; COS3, 3‐year conditional overall survival; ENETS, The European Neuroendocrine Tumor Society; y, year.

### 3.4 CDS and comparison with actual DSS

**Figure 4:**
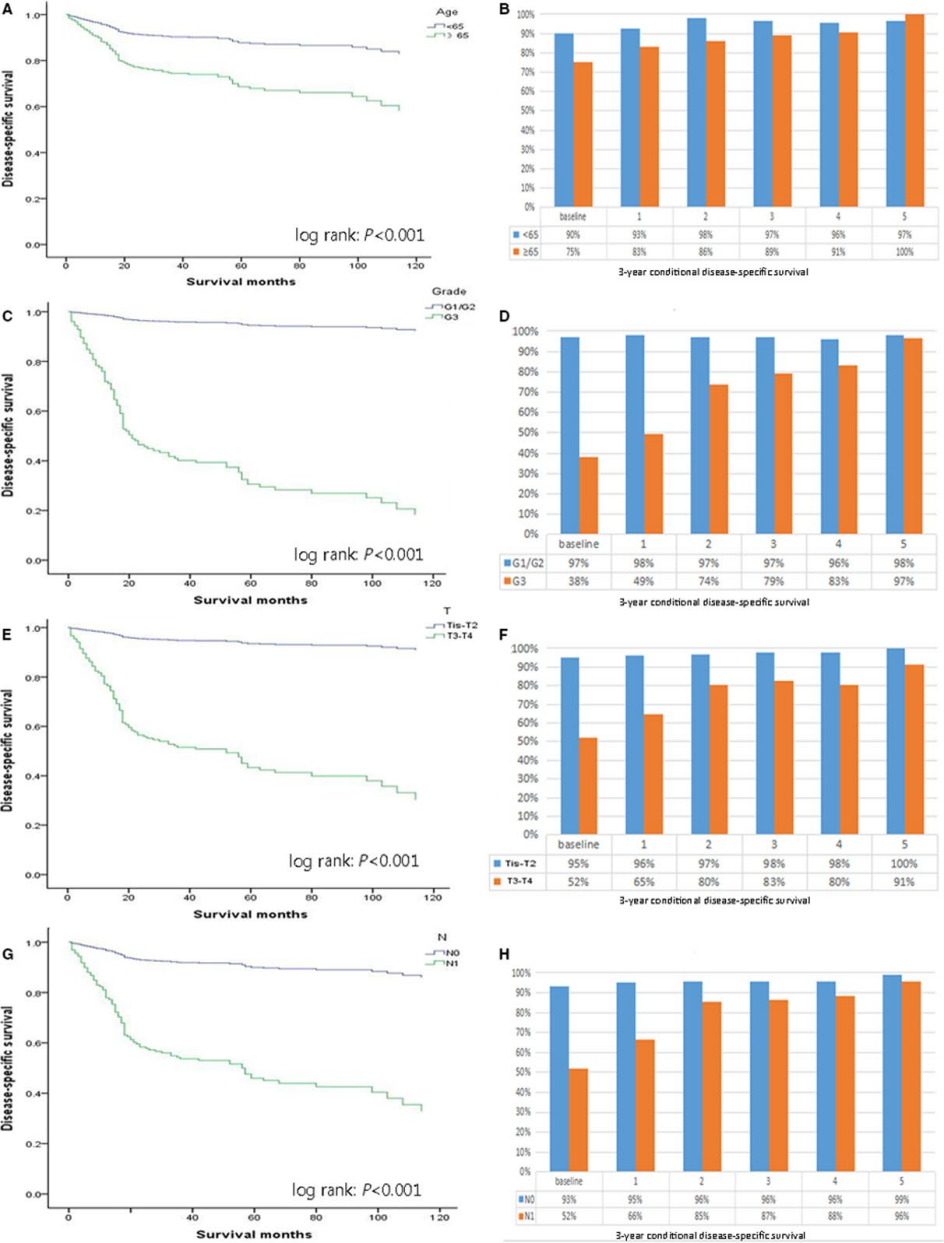
Actual disease‐specific survival stratified by: (A) age, (C) tumor grade, (E) ENETs T stage, and (G) ENETs N stage vs conditional overall survival relative to actual survival stratified by: (B) age, (D) tumor grade, (F) ENETs T stage, and (H) ENETs N stage

The Figure 2B shows the change in the annual actual DSS and CDS3 within 5 years of all patients after surgery. Similar to the trend in COS3, the CDS3 improved with increasing survival time after surgery. With 1‐, 3‐, and 5‐year survivorship, the 3‐year CDS rates improved by +4.4 (89.4%), +9.1 (94.1%), and +12.5 (97.5%), respectively. Moreover, in contrast to the COS3, the CDS3 of all the patients with G‐NETs was more than 90% after 2 years of survival (CDS3 at 2 years = 94.2%). The actual DSS and CDS3 of each prognostic factor at different time points are presented in Figure 4. Notably, for the patients with poorer initial prognosis, the gap between the actual DSS and CDS3 also become increasingly significant. For example, the 8‐year DSS among patients with low‐grade (G3) tumors was only 29.9%, and the CDS3 after 5 years of survival reached 96.8% (Δ66.9%). Nevertheless, the 8‐year actual DSS for patients with high‐grade (G1‐G2) tumors was 93.2%, and the CDS3 at 5 years was 97.9% (Δ4.7%). We also found that when age was included, the differences in CDS3 between the strata of all subgroups decreased with time. For example, the *d* value of CDS3 between the elderly and the young decreased from 0.41 (moderate difference) at the baseline to 0.30 (moderate difference) at 3 years and 0.24 (small difference) at 5 years after surgery.

## 4 DISCUSSION

G‐NETs are rare and account for 0.3%‐1.8% of all gastric cancers.^26^ However, the incidence of NETs of the stomach has increased in recent years.^2^, ^5^ According to the latest American epidemiological survey, there was a 15‐fold increase in the incidence of G‐NETs from 1973 to 2012. However, limited data are available on the clinicopathological features and long‐term outcomes of patients treated for these tumors.

In our large population‐based study of patient with surgically resected G‐NETs, we found that age, tumor grade, T stage, and N stage were independent prognostic factors affecting OS and DSS (all *P* < 0.05) in the multivariate analysis after adjusting for confounding factors. A previous study demonstrated that there are significant differences in the biological behavior and survival of different histological types of G‐NETs. The G‐NETs with poor differentiation (G3) are more aggressive tumors and have poorer prognoses than well‐differentiated (G1‐G2) tumors.^27^, ^28^ The present study confirmed the finding that the actual OS of patients with G3 tumors was worse than the OS of patients with well‐differentiated (G1‐G2) tumors. For example, the 5‐year actual OS of patients with well‐differentiated tumors was 80.4%, which was nearly 4 times the OS of patients with G3 tumors 5 years after surgery. Specifically, the DSS of patients with G3 tumors was significantly poorer than that of patients with well‐differentiated tumors. The 5‐year actual DSS of patients with G1‐G2 tumors (94.7%) was two times greater than that of patients with G3 tumors (31.5%). The ENETS first proposed the staging of G‐NETs according to the TNM system in 2006. The study confirmed that both T stage and N stage are significant factors affecting OS and DSS.

Survival statistics is significantly useful for clinicians in monitoring patients and determining patient prognosis. Usually, the analysis of prognostic factors is on the basis of the traditional survival estimates from previous studies.^28^, ^29^ However, several studies have demonstrated that the risk of death in cancer patients decreases with prolonged postoperative survival time.^30^, ^31^, ^32^ It would be inappropriate to estimate the prognosis of patients who have survived for a period of time after surgery based on the OS and DSS, neglecting the dynamic change in prognosis, as this may cause excessive surveillance. In the CS estimates, “accrued” survival time is considered. Furthermore, using the CS estimate, the clinician can provide clinically relevant survival assessments for patients who have returned to the clinic after their operation inquiring about further prognosis. Our findings demonstrate that not only the OS but also the DSS of G‐NETs change dynamically over time after surgery. There is no doubt that the current study is significant. To our knowledge, this study is the first to define COS and CDS after curative surgery for G‐NETs in a large number of patients.

In the present study, we highlighted several points. No matter how long the survival time, the prognosis of patients improved gradually with each additional year survived after the operation. The results of this study suggest that contrary to the downward trend of the traditional actual OS and DSS, the COS3 and CDS3 improve with increasing survival time after surgery. Specifically, in terms of the DSS, the CDS3 after an additional 2 years of survival in all patients with G‐NETs exceeded 90%, which means that these patients possess a better cancer‐specific life expectancy. Moreover, there is a large difference between CDS and actual DSS estimates among patients initially predicted to have the poorest prognosis. From these survival statistics, we conclude that the effect of these risk factors may decrease over time after surgery. Furthermore, the current data provide more accurate prognostic information for patients who have survived for a period of time after the resection of G‐NETs. Interestingly, data from the current study noted that there is a continuous difference in the hazard risk of COS3 (*d*: 0.62‐0.84) in elderly patients (≥65 years) comparing with their younger counterparts (age < 65 years) with increasing survival time, while the effect of other prognostic factors of COS3 decreased over time. These observations are identical to the hypothesis of the “natural selection effect” first proposed by Zamboni et al.^33^ The author described that most patients with a high risk of malignancy will die soon after surgery and that this gradual death of high‐risk patients facilitates the natural selection of low‐risk patients, making the prognosis of the rest of the patients more favorable. It is helpful for G‐NETs survivors, especially those with initially poor prognosis, to understand the possibility of continued survival over time to ease their anxiety and improve their quality of life.^34^, ^35^ Therefore, more valuable information for follow‐up strategies could be obtained through the CS assessment.

According to the guidelines from the North American Neuroendocrine Tumor Society, the ENETS and the National Comprehensive Cancer Network, a medical history, a physical examination, and an assessment of tumor markers should be included during the 10‐year surveillance. Additionally, imaging examinations such as computed tomography, magnetic resonance imaging, and endoscopic procedures should be included when possible.^19^, ^36^, ^37^ It is well known that there is almost no increase in the CDS3 of all patients after 5 years, even for the patients with the most unfavorable prognosis at the time of surgery, such as those with T3‐T4 stage (CDS3 at 5 years = 91.1%) or N1 stage (CDS3 at 5 years = 95.7%). Early detection of the recurrence of disease is essential for continued treatment. However, the prolongation of surveillance and repeated procedures is burdensome for patients to some extent. To seek more reasonable and effective monitoring programs, there is a need for more stringent long‐term follow‐up prospective studies in the future.

It is difficult to gather large numbers of patients who have a rare tumor with sufficient clinical data. We must admit that our study had several limitations. Based on the SEER database, researchers are provided a unique chance to test the hitherto untested medical hypotheses on an unprecedented amount of patient data. However, there is a potential limitation of this database such as missing dates and misreporting due to the evolving definition of G‐NETs. Such limitations may lead to potential selection bias. To ensure the validity of the follow‐up, patients with detailed information were selected for the analysis. Moreover, the grade of tumor was defined based on the differentiation of the tumor in the SEER database, regardless of the Ki‐67 index, a marker of cellular proliferation, and the mitotic index, which is important for the grading of tumors. Therefore, we could not assign tumor grades to the tumors of the patients in our cohort. Nevertheless, the World Health Organization recommended dividing G‐NETs into G1, G2, and G3 on the basis of differentiation, which has been shown to have prognostic significance independent of tumor stage.^38^ In addition, other endpoints besides death, such as time to recurrence and disease‐free survival, were not analyzed because of the absence of disease recurrence data in the SEER database. We evaluated both the OS and DSS, which resolves difficulties in retrospectively determining the cause of death and the possibility of “cause of‐death‐interpretation bias” as described by Machtay et al.^39^

In conclusion, age, tumor grade, T stage, and N stage were the clinicopathological factors significantly associated with the prognosis of patients with G‐NETs. The prognosis of G‐NETs survival improves gradually with each year of survival after surgery. The CDS3 of all patients was more than 90% across all independent prognostic factors after 5 years of survival, which means that most patients with G‐NETs have excellent results. Furthermore, it would be useful for clinicians and researchers to provide more accurate clinically relevant survival assessments to patients using the CS estimate, to allow them to make life plans and to monitor the disease intensity during follow‐up after a G‐NETs diagnosis.

## DISCLOSURES

The authors made no disclosures.
