# Supplementary figures and images for: Clinicopathological features and prognosis of patients with gastric neuroendocrine tumors: A population‐based study
Source: Cancer Med. 2018 Oct 11;7(11):5359–69. doi: 10.1002/cam4.1683 (PMC6246951; doi:10.1002/cam4.1683)

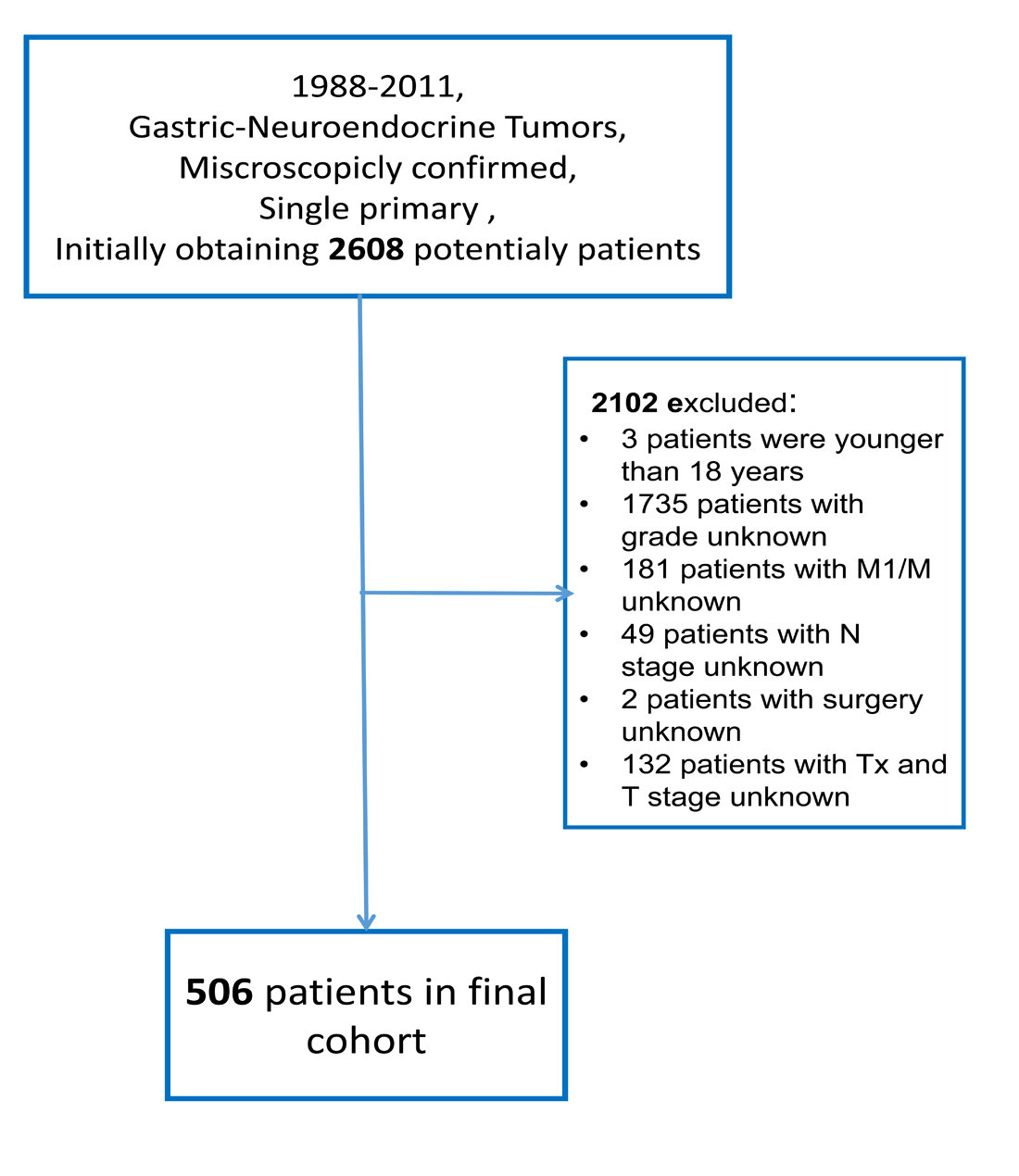

Supplement: Supplementary file 1 [file CAM4-7-5359-s001.tif]
