# Supplementary material for: Clinicopathological features and prognosis of patients with gastric neuroendocrine tumors: A population‐based study
Source: Cancer Med. 2018 Oct 11;7(11):5359–69. doi: 10.1002/cam4.1683 (PMC6246951; doi:10.1002/cam4.1683)
Supplement: Supplementary file 2 [file CAM4-7-5359-s002.docx]

**Supplemental Table 1. Category Parameters for Census-Based Socioeconomic Status Variables**

| **Variable** | | **Advantaged** | **Disadvantaged** |
| --- | --- | --- | --- |
| Education | (% < High school education ACS 2011-15) | 4.77-13.89 | 13.95-33.66 |
| Poverty | (% Families below poverty ACS 2011-15) | 2.89-11.36 | 11.82-32.34 |
| Unemployment | (% Unemployed ACS 2011-15) | 2.15-8.70 | 8.77-17.41 |
| Family income | (Median family income, $ ACS 2011-15) | 64150-125990 | 33640-64080 |
| Foreign-born | (Foreign-born people, % ACS 2011-15) | 20.08-41.91 | 0.22-19.07 |
| Language isolation | (Households living in language isolation, %ACS 2011-15) | 6.38-21.34 | 0.00-6.29 |

Abbreviation: ACS, American Community Survey.

**Supplemental Table 2. Univariate Analysis of Prognostic Factors of Overall Survival and Disease-Specific Survival for Gastric Neuroendocrine Tumor Patients**

|  | **Overall Survival** | | | |  | **Disease-Specific Survival** | | | |
| --- | --- | --- | --- | --- | --- | --- | --- | --- | --- |
| **Variable** | **HR** | **95%CI** | | **P** |  | **HR** | **95%CI** | | **P** |
| **Age y** |  |  |  | <0.001 |  |  |  |  | <0.001 |
| <65 | Ref. |  |  |  |  | Ref. |  | |  |
| ≥65 | 3.92 | 2.87-5.35 | | <0.001 |  | 2.89 | 1.90-4.38 | | <0.001 |
| **Sex** |  |  |  | 0.003 |  |  |  |  | 0.006 |
| Female | Ref. |  | |  |  | Ref. |  | |  |
| Male | 1.58 | 1.17-2.12 | | 0.003 |  | 1.78 | 1.18-2.69 | | 0.006 |
| **Race** |  |  |  | 0.301 |  |  |  |  | 0.151 |
| White | Ref. |  |  |  |  | Ref. |  | |  |
| Black | 1.37 | 0.90-2.09 | | 0.141 |  | 1.28 | 0.71-2.31 | | 0.421 |
| Others | 1.39 | 0.81-2.37 | | 0.232 |  | 2.08 | 1.10-3.93 | | 0.024 |
| Unknown | 0.55 | 0.08-3.93 | | 0.551 |  | <0.001 | 0.00-3.64 | | 0.964 |
| **Ethnicity** |  |  |  | 0.018 |  |  |  |  | 0.388 |
| Spanish-Hispanic-Latino | Ref. |  | |  |  | Ref. |  | |  |
| Non-Spanish-Hispanic-Latino | 0.59 | 0.38-0.91 | | 0.018 |  | 0.78 | 0.45-1.36 | | 0.388 |
| **Primary site** |  |  |  | <0.001 |  |  |  |  | <0.001 |
| Proximal | Ref. |  |  |  |  | Ref. |  |  |  |
| Middle and Distal | 0.48 | 0.32-0.70 | | 0.000 |  | 0.32 | 0.19-0.53 | | <0.001 |
| Overlapping lesions | 0.96 | 0.45-2.03 | | 0.916 |  | 1.00 | 0.42-2.39 | | 0.995 |
| Stomach, NOS | 0.52 | 0.36-0.76 | | 0.001 |  | 0.34 | 0.21-0.57 | | <0.001 |
| **Size, mm** |  |  |  | <0.001 |  |  |  |  | <0.001 |
| ≤20 | Ref. |  |  |  |  | Ref. |  |  |  |
| >20 | 3.49 | 2.50-4.88 | | <0.001 |  | 8.61 | 5.00-14.83 | | <0.001 |
| Unknown | 2.41 | 1.59-3.66 | | <0.001 |  | 4.64 | 2.41-8.92 | | <0.001 |
| **Grade** |  |  |  | <0.001 |  |  |  |  | <0.001 |
| G1-G2 | Ref. |  | |  |  | Ref. |  | |  |
| G3 | 5.98 | 4.43-8.07 | | <0.001 |  | 21.15 | 12.84-34.81 | | <0.001 |
| **ENETS T stage** |  |  |  | <0.001 |  |  |  |  | <0.001 |
| Tis-T2 | Ref. |  | |  |  | Ref. |  | |  |
| T3-T4 | 3.75 | 2.79-5.05 | | <0.001 |  | 12.46 | 7.76-20.00 | | <0.001 |
| **ENETS N stage** |  |  |  | <0.001 |  |  |  |  | <0.001 |
| N0 | Ref. |  | |  |  | Ref. |  | |  |
| N1 | 3.16 | 2.32-4.29 | | <0.001 |  | 7.35 | 4.85-11.15 | | <0.001 |
| **Chemotherapy** |  |  |  | <0.001 |  |  |  |  | <0.001 |
| No | Ref. |  | |  |  | Ref. |  | |  |
| Yes | 4.15 | 2.95-5.85 | | <0.001 |  | 9.28 | 6.12-14.09 | | <0.001 |
| **Radiation** |  |  |  | <0.001 |  |  |  |  | <0.001 |
| No | Ref. |  | |  |  | Ref. |  | |  |
| Yes | 4.00 | 2.62-6.10 | | <0.001 |  | 6.70 | 4.16-10.80 | | <0.001 |
| **Marital status** |  |  |  | 0.346 |  |  |  |  | 0.237 |
| Unmarried | Ref. |  |  |  |  | Ref. |  |  |  |
| Married | 0.84 | 0.62-1.14 | | 0.260 |  | 1.24 | 0.80-1.92 | | 0.340 |
| Unknown | 0.63 | 0.29-1.38 | | 0.248 |  | 0.42 | 0.10-1.77 | | 0.238 |
| **Education** |  |  |  | 0.658 |  |  |  |  | 0.977 |
| Advantaged | Ref. |  | |  |  | Ref. |  | |  |
| Disadvantaged | 0.94 | 0.70-1.26 | | 0.658 |  | 0.99 | 0.66-1.50 | | 0.977 |
| **Poverty** |  |  |  | 0.626 |  |  |  |  | 0.944 |
| Advantaged | Ref. |  | |  |  | Ref. |  | |  |
| Disadvantaged | 1.08 | 0.80-1.45 | | 0.626 |  | 1.02 | 0.67-1.53 | | 0.944 |
| **Unemployment** |  |  |  | 0.690 |  |  |  |  | 0.711 |
| Advantaged | Ref. |  | |  |  | Ref. |  | |  |
| Disadvantaged | 1.06 | 0.79-1.43 | | 0.690 |  | 1.08 | 0.72-1.63 | | 0.711 |
| **Family income** |  |  |  | 0.477 |  |  |  |  | 0.733 |
| Advantaged | Ref. |  | |  |  | Ref. |  | |  |
| Disadvantaged | 1.11 | 0.83-1.50 | | 0.477 |  | 0.93 | 0.62-1.40 | | 0.733 |
| **Foreign-born** |  |  |  | 0.467 |  |  |  |  | 0.819 |
| Advantaged | Ref. |  | |  |  | Ref. |  | |  |
| Disadvantaged | 1.12 | 0.83-1.50 | | 0.467 |  | 0.95 | 0.63-1.44 | | 0.819 |
| **Language isolation** |  |  |  | 0.446 |  |  |  |  | 0.940 |
| Advantaged | Ref. |  | |  |  | Ref. |  | |  |
| Disadvantaged | 1.12 | 0.83-1.50 | | 0.446 |  | 0.98 | 0.65-1.48 | | 0.940 |

Abbreviation: Y, Year; NOS, Not otherwise specified; ENETS, The European Neuroendocrine Tumor Society; Unmarried: Single (never married), Separated Divorced, Widowed, Unmarried or domestic partner (same sex or opposite sex or unregistered).

**Supplemental Table 3. Overall Survival of the Patients with Gastric Neuroendocrine Tumors in Relation to Clinicopathological Factors**

| **Patient Survival, %** | | | | | | | | | |
| --- | --- | --- | --- | --- | --- | --- | --- | --- | --- |
| **Characteristic** | 1y | 2y | 3y | 4y | 5y | 6y | 7y | 8y | P Value |
| Overall | 90.2% | 80.8% | 77.3% | 73.9% | 68.8% | 64.8% | 62.7% | 58.9% |  |
| **Age y** |  |  |  |  |  |  |  |  | <0.001 |
| <65 | 95.1% | 89.1% | 87.4% | 85.5% | 82.1% | 80.1% | 78.3% | 78.3% |  |
| ≥65 | 82.7% | 67.8% | 61.7% | 55.9% | 48.3% | 41.4% | 39.1% | 30.8% |  |
| **Grade** |  |  |  |  |  |  |  |  | <0.001 |
| G1-G2 | 95.7% | 92.1% | 89.0% | 85.5% | 80.4% | 76.1% | 74.0% | 70.5% |  |
| G3 | 70.1% | 39.0% | 34.3% | 31.3% | 27.1% | 24.5% | 22.8% | 19.6% |  |
| **ENETS T stage** |  |  |  |  |  |  |  |  | <0.001 |
| Tis-T2 | 94.7% | 89.9% | 86.4% | 82.7% | 78.6% | 74.6% | 73.2% | 68.7% |  |
| T3-T4 | 76.4% | 52.7% | 49.4% | 46.8% | 39.8% | 36.0% | 32.6% | 30.8% |  |
| **ENETS N stage** |  |  |  |  |  |  |  |  | <0.001 |
| N0 | 94.7% | 88.4% | 85.0% | 81.5% | 76.0% | 71.8% | 69.8% | 65.7% |  |
| N1 | 73.1% | 51.8% | 47.9% | 44.9% | 41.5% | 38.3% | 36.1% | 33.3% |  |

Abbreviation: y, year; ENETS, The European Neuroendocrine Tumor Society.

**Supplemental Table 4. Disease-Specific Survival of the Patients with Gastric Neuroendocrine Tumors in Relation to Clinicopathological Factors**

| **Patient Survival, %** | | | | | | | | | |
| --- | --- | --- | --- | --- | --- | --- | --- | --- | --- |
| **Characteristic** | 1y | 2y | 3y | 4y | 5y | 6y | 7y | 8y | P Value |
| Overall | 93.9% | 86.1% | 84.5% | 84.0% | 80.9% | 79.9% | 79.3% | 79.3% |  |
| **Age y** |  |  |  |  |  |  |  |  | <0.001 |
| <65 | 96.7% | 91.3% | 90.2% | 90.2% | 88.7% | 87.2% | 86.2% | 86.2% |  |
| ≥65 | 89.4% | 77.5% | 75.1% | 73.6% | 67.5% | 67.5% | 67.5% | 67.5% |  |
| **Grade** |  |  |  |  |  |  |  |  | <0.001 |
| G1-G2 | 99.5% | 98.1% | 97.3% | 97.0% | 94.7% | 94.1% | 93.2% | 93.2% |  |
| G3 | 73.3% | 41.6% | 37.5% | 36.4% | 31.5% | 29.9% | 29.9% | 29.9% |  |
| **ENETS T stage** |  |  |  |  |  |  |  |  | <0.001 |
| Tis-T2 | 98.9% | 96.1% | 95.2% | 94.9% | 93.4% | 92.7% | 92.7% | 92.7% |  |
| T3-T4 | 78.6% | 55.7% | 52.2% | 51.2% | 44.6% | 43.1% | 41.1% | 41.1% |  |
| **ENETS N stage** |  |  |  |  |  |  |  |  | <0.001 |
| N0 | 98.2% | 94.2% | 93.1% | 92.8% | 90.0% | 89.4% | 88.6% | 88.6% |  |
| N1 | 77.3% | 54.8% | 51.7% | 50.6% | 46.7% | 44.9% | 44.9% | 44.9% |  |

Abbreviation: y, year; ENETS, The European Neuroendocrine Tumor Society.
